# On the Electrophilic Character of Molecules Through Its Relation with Electronegativity and Chemical Hardness

**DOI:** 10.3390/ijms13022160

**Published:** 2012-02-17

**Authors:** Nazmul Islam, Dulal C. Ghosh

**Affiliations:** Department of Chemistry, University of Kalyani, Kalyani 741235, West Bengal, India; E-Mail: nazmul.islam786@gmail.com

**Keywords:** electronegativity, hardness, electrophilicity index, electronegativity equalization principle, hardness equalization principle, electrophilicity equalization principle

## Abstract

Electrophilicity is an intrinsic property of atoms and molecules. It probably originates logistically with the involvement in the physical process of electrostatics of soaked charge in electronic shells and the screened nuclear charge of atoms. Motivated by the existing view of conceptual density functional theory that similar to electronegativity and hardness equalization, there should be a physical process of equalization of electrophilicity during the chemical process of formation of hetero nuclear molecules, we have developed a new theoretical scheme and formula for evaluating the electrophilicity of hetero nuclear molecules. A comparative study with available bench marking reveals that the hypothesis of electrophilicity and equalization, and the present method of evaluating equalized electrophilicity, are scientifically promising.

## 1. Introduction

Electrophilicity is a very useful theoretical construct of conceptual chemistry originating from the fruition of the long effort of understanding the mechanisms of organic reactions [1]. A molecule can be theoretically dissected into a Lewis acid and a Lewis base, and the formation of the molecule can be conceived as a reaction between an acid and a base or between an electrophile and a nucleophile. In general, the electrophiles are electron lovers or electron deficient and hence prefer to accept electrons and form bonds with nucleophiles. Thus electrophilicity is a useful structural depictor of reactivity and is frequently used in the analysis of the chemical reactivity of molecules [1–12].

Ingold [13] proposed the first global electrophilicity scale to describe electron-deficient (electrophile) and electron-rich (nucleophile) species based on the valence electron theory of Lewis. Electrophilicity is the intrinsic structural property of being an electrophile. Sporadic information has appeared regarding electrophiles and electrophilicity in conceptual theoretical chemistry and several methods exist to rank electrophiles in order of philicity or reactivity [3]. The present day theoretical paradigm of chemistry—the conceptual density functional theory, CDFT [12,14–18] has introduced three magic words—electronegativity, chemical hardness and electrophilicity to chemistry and physics.

Although various definitions and scales of measurement of electronegativity and hardness are known, there has been hardly any effort made to understand the fundamental nature of electrophilicity from first principles. There have been several empirical efforts to rank the electrophiles in order of their reactivity in terms of equilibrium constants of chemical reactions [19,20]. The quantitative definition of electrophilicity was put forward by Parr and co-workers [10] following the work of Maynard *et al*. [11].

Parr *et al*. [10] defined global electrophilicity as a quantitative intrinsic numerical value and suggested the term electrophilicity index, *ω*, a new global reactivity descriptor of atoms and molecules, as

(1) $$\omega =\frac{\mu^{2}}{2\eta }$$

where *μ* is the chemical potential and *η* is the hardness of the system.

Thereafter there was a surge of research on electrophilicity [1–9,12].

Since electronegativity, *χ* is additive, inverse of the chemical potential, the electrophilicity index, *ω*, can be written as

(2) $$\omega =\frac{\chi^{2}}{2\eta }$$

Electrophilicity is a property of atoms which signifies the energy lowering process on soaking electrons from donors. The electrophilicity index measures the stabilization in energy when the system acquires an additional electronic charge from the environment. In fact Chaquin [21] has drawn an analogy between electrophilicity and electrical power which has the classical equation as *P* = *V*^2^/*R*, where *R* is resistance and *V* is voltage. In this sense the electrophilicity index is a kind of power.

The Equation 2 for electrophilicity physically means that it simultaneously encompasses both the properties of the electrophile to acquire an additional electronic charge driven by *μ*^2^ and the resistance of the system to exchange electronic charge with the environment described by *η*. However, effectively it is conceived as representing the stabilization energy of the system when it becomes saturated by electrons coming from the surroundings.

The fundamental nature and operational significance of electrophilicity have been critically analyzed by Gazquez [22].

Parr *et al.* [10] evaluated *ω* in terms of Equation 2 by invoking the operational and approximate formula of *χ* and *η* suggested by Parr, Pearson and others [23,24]. Ayers *et al.* [25,26] seem to furnish a critical justification for measuring *ω* in terms of the ansatz in Equation 2. The chemical potential and chemical hardness are key indicators of the overall reactivity of the molecule and are the most fundamental descriptors of charge transfer during a chemical reaction. Hence, it is not surprising that such indicators can usually be written as functions of chemical potential and chemical hardness. Ayers *et al*. [25,26] have further opined that chemical potential alone cannot be a measure of electrophilicity: Whereas a molecule with low chemical potential is a good electrophile, an extremely hard molecule has feeble electron acceptability. Consequently, a measure of molecular electrophilicity depends on both the chemical potential and the chemical hardness.

The evaluation of *ω* requires the theoretical or experimental ionization energy, *I* and electron affinity, *A* of atoms and molecules. But the experimental *I* and *A* for any chemical system are still undetermined. The theoretical evaluation of these descriptors invoking Hartree-Fock SCF theory and Koopman’s theorem is an unsuccessful and yet unresolved venture of theoretical chemistry [27–30]. Therefore, we, are seeking some alternative algorithm, semi-empirical in nature, to evaluate the density functional descriptors, without the experimental or theoretical *I* and *A* of chemical systems. We have demonstrated [31–38] that the nature of electronegativity, hardness and the electrophilicity index are fundamentally qualitative *per se* as they are not observable. Thus these descriptors are noumena—that is to say, they occur but cannot be seen. Hence, the possibility of experimental determination of such descriptors is ruled out. And since these descriptors are not observable, no quantum mechanical operators can be suggested for them. This rules out any quantum mechanical evaluation of such descriptors.

It is important to mention here some outstanding work of Putz and his coworkers [39–47] on electronegativity and hardness and their usefulness for the theoretical prediction of several physicochemical properties—such as the fundamentals of chemical bonding. The basic physico-chemical concepts of density functional theory are employed by Putz *et al*. [39–47] to highlight the role of energy in chemical structure, while its extended influence in electronic localization function helps in the understanding of chemical bonding. In this context the energy functionals accompanied by electronic localization functions may provide a comprehensive description of the global-local levels of electronic structures in general and of chemical bonds in particular. It has been shown that the aromaticity of a peripheral topological path may be well described by superior finite difference schemes of electronegativity and chemical hardness indices under certain calibrating conditions. They [39–47] have also discussed at length the problem of observability to electronegativity and chemical hardness. Invoking a semi classical method, Putz introduced the electronegativity of an element as the power by which the frontier electrons are attracted to the center of the atom, this being a stability measure of the atomic system as a whole. A new chemical hardness expression in terms of atomic radius has also been given by Putz *et al*. [39–47]. A unified Mulliken valence with Parr groundstate electronegativity picture has been presented by Putz and his coworkers [39–47].

One may think logistically that it is quite possible that the electronic structure, especially the shell structure and the physical process of screening of the nuclear charge of atoms, are intimately linked to the origin and development of the hardness, electronegativity and electrophilicity of atoms.

Hence, this tendency of charge soaking and energy lowering must involve attraction between the screened nucleus and the electronic charge in the shells of the atoms., Therefore, it transpires that shell structure and the screened nuclear charge of the atoms act conjointly to develop the new electrostatic property—the electrophilicity of atoms. Because the soaked electron density must be accommodated in the shells/sub-shells, energy is necessarily released by the electrostatic attraction of the nuclei. Relying upon the above conjecture of the mechanism of development of electrophilicity, we [38] have proposed an electrostatic approach to arrive at a new formula of evaluating *ω* of atoms in terms of their most probable radii, the size descriptors.

We [31–37] have posited that there is much conceptual commonality between the two fundamental theoretical descriptors of chemistry and physics—the electronegativity and the hardness and both the fundamental descriptors originate from the same source—the electron attracting power of the screened nucleus upon the valence electrons. In a recent work [38], we derived a new formula for evaluating the electrophilicity index *ω* based on the hypothesis that hardness and electronegativity originate from the same source in the structure of atoms and that they must be proportional to each other *i.e.*,

(3) χ∞η

or,

(4) χ=L·η

where *L* is the proportionality constant.

Putting *χ* = *L η* in the Equation 2 we get

(5) $$\omega =\frac{L^{2}\eta^{2}}{2\eta }=\frac{L^{2}\eta }{2}$$

Now classically, the energy *E*(*N*) of charging a conducting sphere of radius *r* with charge *q* is given by [48]

(6) $$E(N)=\frac{q^{2}}{2r}\;(\text{in C}.\text{G}.\text{S}.\text{Unit})$$

In Equation 6,*E*(*N*) is in ergs, *q* is the charge in electrostatic units and *r* is in cm.

Now, for an atom, the change in energy associated with the increase or decrease of *q* can be estimated in terms of Equation 6. In particular, on removal of an electron of charge, *e* to make the charge (*q* − *e*), the energy change would be the ionization energy, *I*. Similarly, the energy evolved on addition of an electron with *q* (*q* + *e*) would be the electron affinity, *A*. Hence,

(7) $$I=E(N+1)-E(N)=\left[\frac{{\left(q+e\right)}^{2}}{2r}-\frac{q^{2}}{2r}\right]$$

and

(8) $$A=E(N)-E(N-1)=\left[\frac{q^{2}}{2r}-\frac{{\left(q-e\right)}^{2}}{2r}\right]$$

Now putting the values of *I* and *A* from above into the formula of global hardness of Parr and Pearson [24], we get

(9) $$\eta =\frac{\left(I-A\right)}{2}=\left[\left\{\frac{{\left(q+e\right)}^{2}}{2r}-\frac{q^{2}}{2r}\}-\{\frac{q^{2}}{2r}+\frac{{\left(q-e\right)}^{2}}{2r}\right\}\right]$$

or,

(10) $$\eta =\frac{e^{2}}{2r}$$

where *e* is the electronic charge in esu and *r* is the most probable or absolute radius of the atom in cm.

Equation 10 clearly shows that hardness has the dimension of energy.

Now as the Parr and Pearson’s formula of hardness is approximate, we [49] therefore proposed that the hardness, *η* is not exactly equal to *e*^2^/2*r*, rather, in all probability, proportional to *e*^2^/2*r*.

(11) $$\eta \infty \frac{e^{2}}{2r}$$

(12) $$\text{or},\;\eta =C\frac{e^{2}}{2r}$$

where *C* is the proportionality constant.

Comparing Equations 5 and 12 we get,

(13) $$\omega =\frac{L^{2}{Ce}^{2}}{r}$$

Since, *L* and *C* are constants, we can write

(14) $$\omega \propto e^{2}/r$$

The new formula for evaluating *ω* is

(15) $$\omega \;(\text{eV})=\frac{{Ke}^{2}}{r}$$

where *K* is the proportionality constant, *e* is the electronic charge and *r* is the most probable radii of atoms.

## 2. Electrophilicity Equalization Principle

The electrophilicity equalization principle, similar to electronegativity equalization and hardness equalization, is implicit and sporadically segregated in the literature of CDFT. However, we have found that there are adherents and detractors of the electrophilicity equalization principle. A survey of the literature shows that several workers [50,51] have studied the variation of electrophilicity during molecular vibrations and internal rotations and it has been found [50] that under constant chemical potential and *V*(*r*), there would be a minimum electrophilicity principle along a reaction path. Chaquin [21], by analogy with classical electrostatics, suggests an interpretation of Parr’s “electrophilicity index” as a “global energy index” leading to a “minimum electrophilicity principle”. It is expected to decrease during an exothermal process and in comparison with the principle of maximum hardness, the “principle of minimum electrophilicity” seems to be more often obeyed [52–54]. It is pertinent to mention here the work of Ayers and Parr on hardness and hyper hardness equalization [25,26]. According to them [25,26], since electronegativity and hardness are both equalized, the electrophilicity (being the ratio of the two) must also be equalized. The electrophilicity equalization principle is also implicit in the work of Chaquin [21] and Noorizadeh and Shakerzadeh [52,53]. Therefore, it is quite probable that there should be, similar to the physical process of electronegativity equalization [23,55] and the hardness equalization [25,26,34–37,56–58], an analogous process of equalization of electrophilicity during the event of molecule formation. Looking at the Equation 2, for the definition of electrophilicity, it is given that electrophilicity is the result of conjoint action of two global quantities of CDFT, the electronegativity and the chemical hardness. Thus the strongest argument in favour of electrophilicity equalization follows from the fact that, since electronegativity equalization is unequivocal and widely accepted and hardness equalization is also now established, and since if electronegativity and hardness are both equalized, then electrophilicity (being their ratio) must also be inevitably equalized. Thus, the present analysis logistically establishes that it is unequivocal that electrophilicity equalization exists and is manifest during the chemical events of molecule formation. It is worth noting that the electrophilicity equalization principle was challenged by von Szentpaly [59] who has ruled out any possibility of electrophilicity equalization. But our present analysis logistically establishes that it is unequivocal that electrophilicity equalization exists and is manifest during the chemical events of molecule formation.

Recently, Chattaraj *et al*. [8] have suggested a theoretical method of computing an equalized electrophilicity index on molecule formation. However, he assumed that the hardness and the electronegativity act separately in opposite directions while producing a new property—electrophilicity and its equalization. Moreover, in order to compute the equalized electrophilicity indices of molecules, they invoked the geometric mean principle of electronegativity equalization [55] and hardness equalization [58]. However, we believe that the method of computing equalized electrophilicity index of Chattaraj *et al*. [8] is not acceptable, in view of earlier findings that the geometric as well as the other mean principle of hardness equalization has not been that successful in studying chemical interactions [35].

This method of Chattaraj has been contradicted by von Szentpaly [59]. Szentpaly in a recent communication [59] ruled out the possibility of electrophilicity equalization and also investigated the geometric mean equalization model proposed by Chattaraj *et al*. [8]. He showed that there is no ground for suggesting a principle of electrophilicity equalization by arithmetic, geometric, or harmonic averaging of atomic values. We also partially support Szentpaly because we believe that the theoretical model and mechanism of the process of electrophilicity equalization put forward by Chattaraj *et al*. [8] is not convincing on theoretical consideration of the development and origin of hardness, electronegativity and electrophilicity. We have also pointed out the theoretical discrepancies apparent in the method suggested by Chattaraj *et al*. [8] in invoking the mean principle. Similar to Szentpaly,, we also do not rely upon the various mean principles in order to point out the theoretical discrepancy in the method of Chattaraj under this reference,. Thus, the physical process of electrophilicity equalization through the simple consideration of the geometric mean of atomic electronegativity and hardness does not seem to be a convincing proposition, considering that we have convincingly demonstrated the inadequacy of geometric mean principle during the hardness equalization process [34].

After publication of the paper of Szentpaly [59], Chattaraj *et al*. [60] commented on the possibility of ruling out any electrophilicity equalization principle and tried to justify the electrophilicity equalization principle. In the next communication Szentpaly [61] further criticized the electrophilicity equalization principle.

In contrast to Szentpaly [59], we [34–38] believe that the equalization process works in the formation of molecules but we agree with the idea that the geometric as well as the other mean principle of hardness equalization is not particularly successful for studying chemical interactions and hence to study equalization of descriptors. We consider electrophilicity to be one of the most important properties/descriptors of atoms and molecules. In this report, we have developed a new scheme for the physical process of electrophilicity equalization during the chemical event of formation of hetero nuclear molecules.

## 3. Method of Computation

Our present work is based upon the hypothesis of electrophilicity equalization with the aim to suggest a formula for evaluating the equalized electrophilicity of a molecule in terms of the atomic electrophilicities.

Let us consider the formation of a polyatomic molecule ABC… from its constituents. The polyatomic molecule is assumed to be a cluster of atoms where one atom is at the center and the others are surrounding it. Let us assume that the central atom is A and the ligands surrounding the central atom are B, C, … as represented below:

(16) A+B+C+…→ABC…

Let us consider the electrophilicity index of the molecule and the combining atoms are *ω*_M_ and *ω*_B_, *ω*_C_ *ω*_n_, respectively.

Let us further assume that *r*_A_ is the most probable atomic radius of the central atom A and *r*_B_, *r*_C_, *r*_n_ are the most probable atomic radii of the ligands B, C, … n, respectively.

It is the result of rigorous investigation of the status and the physical condition of atoms in molecules that the atoms remain in a slightly modified state in the molecule [62–64]. Since the radii of atoms in any molecule are not available and since there is no hint of any method for evaluation of the radius of any atom as part of any molecule, we can therefore safely assume, for all approximate purposes, that the radius of the atom in a polyatomic molecule is approximately equal to its most probable radius. Now, during the formation of the poly atomic molecule, let *δ* be the total amount of charge transfer from the central atom A to n, the number of the ligands surrounding the central atom. The total amount of charge transferred (*δ*) is distributed among the ligands and, of course, the charge distribution is governed by the electrophilicity indices of the individual ligands.

Let, B, C, … nth ligands have the charges *δ*_1_, *δ*_2_, … *δ*_n_ respectively in the molecular cluster and let

(17) $$\delta =\delta_{1}+\delta_{2}+\ldots +\delta_{n}$$

Now, after the charge transfer and invoking Equation 3 above, the electrophilicity indices of the central atom A in the poly atomic molecule becomes

(18) $${\omega^{/}}_{\text{A}}=\frac{K{\left(e-\delta \right)}^{2}}{2r_{A}^{/}}$$

and the electrophilicity index of the ligands in the molecule becomes

(19) $$\begin{array}{l} {\omega^{/}}_{\text{B}}=\frac{K{\left(e+\delta_{1}\right)}^{2}}{2r_{B}^{/}},\\ {\omega^{/}}_{\text{C}}=\frac{K{\left(e+\delta_{2}\right)}^{2}}{2r_{C}^{/}},\\ {\omega^{/}}_{\text{n}}=\frac{K{\left(e+\delta_{n}\right)}^{2}}{2r_{n}^{/}} \end{array}$$

respectively, where *r*^/^_A_, *r*^/^_B_, *r*^/^_C_, … *r*^/^_n_ are the radii of atoms in the molecule. Similarly *ω*^/^_A,_ *ω*^/^_B_, *ω*^/^_C_, …*ω*^/^_n_ are the electrophilicity indices of the atoms in the molecule.

Expanding Equation 18, (*e* − *δ*)^2^, and neglecting the *δ*^2^ term in the expansion we get: The electrophilicity index of the central atom, A as

(20) $${\omega^{/}}_{\text{A}}=\frac{K(e^{2}-2e\delta )}{2r_{A}^{/}}$$

and similarly expanding Equation 19, (*e* + *δ*)^2^ and neglecting the square terms from the expansion of electrophilicity indices of the ligands in the molecule, the formulae for electrophilicity indices of atoms in the molecule appear as

(21) $${\omega^{/}}_{\text{n}}=\frac{K(e^{2}-2e\delta )}{2r_{A}^{/}}$$

The electrophilicity equalization principle implies that, after the formation of the molecule, the electrophilicity indices of the individual constituents must be equalized, *i.e.*,

(22) $$\omega_{\text{M}}={\omega^{/}}_{\text{A}}={\omega^{/}}_{\text{B}}={\omega^{/}}_{\text{C}}=\ldots ={\omega^{/}}_{\text{n}}$$

The Equation 23 implies

(23) $$\begin{array}{l} \omega_{\text{M}}=\frac{K(e^{2}-2e\delta )}{2r_{\text{A}}^{/}}=\frac{K(e^{2}+2e\delta_{1})}{2r_{\text{B}}^{/}}=\frac{K(e^{2}+2e\delta_{2})}{2r_{\text{C}}^{/}}=\ldots =\frac{K(e^{2}+2e\delta_{n})}{2r_{\text{n}}^{/}}\\ =\frac{K\{(e^{2}-2e\delta )+(e^{2}+2e\delta_{1})+(e^{2}+2e\delta_{2})+\ldots +(e^{2}+2e\delta_{\text{n}})\}}{\left(2{r^{/}}_{\text{A}}+2{r^{/}}_{\text{B}}+2{r^{/}}_{\text{C}}+\ldots +2{r^{/}}_{\text{n}}\right)}\\ =\frac{K(e^{2}-2e\delta +{\text{n}e}^{2}+2e\delta )}{\left(2{r^{/}}_{\text{A}}+2{r^{/}}_{\text{B}}+2{r^{/}}_{\text{C}}+\ldots +2{r^{/}}_{\text{n}}\right)}\\ =\frac{K(e^{2}+\text{n}e^{2})}{\left(2{r^{/}}_{\text{A}}+2{r^{/}}_{\text{B}}+2{r^{/}}_{\text{C}}+\ldots +2{r^{/}}_{\text{n}}\right)} \end{array}$$

In the reverse process, where charge transfer from the ligands to the central atom occurs, the same formula results.

Invoking the approximation that atoms retain their identity in the molecule [62–64], we can replace the *r*^/^term by the most probable radii of the corresponding atom in Equation 24 and it finally appears as

(24) $$\omega_{\text{M}}=\frac{K\;(n+1)e^{2}}{2\sum_{i}r_{i}}$$

Equation 25 computes electrophilicity index in esu, and in electron volts it appears as:

(25) $$\omega_{\text{M}}=\frac{K\;7.2(n+1)}{\sum_{i}r_{i}}$$

where, *r_i_* is the atomic radius in Angstrom units.

We have calculated the standardized value of *K* = 0.382516 (for diatomic molecules) and *K* = 0.172 (for poly atomic molecules). To compute *K* for hetero nuclear diatomics, we have proceeded as follows. First the geometry optimization of the corresponding molecules has been furnished using the 6-31G* basis set of the Hyperchem 8.0 professional program [65] to compute the HOMO and LUMO energies of the molecule. After that, using Koopman’s approximation we have computed the *I*’s and *A*’s of the molecules. Thereafter considering the formula of Parr *et al.* [10,24] we have computed the electrophilicity indices data of the molecules and labeled it as *ab initio* electrophilicity indices data of the molecules. Thereafter, we have divided the *ab initio* theoretically computed electrophilicity indices data of the molecules by $\frac{7.2(n+1)}{\sum_{i}r_{i}}$. Then, we have taken the mean of several K’s and the mean value obtained is 0.278. To compute the K for polyatomic molecules, we have divided global electrophilicity indices of some poly-atomic molecules, computed using the experimental Ionization energy and electron affinity of the corresponding molecule [66] and adopting the formula of *ω* of Parr *et al*. [10], by $\frac{7.2(n+1)}{\sum_{i}r_{i}}$. Thereafter, we have taken the mean of several *K*’s and the mean value obtained is 0.172. In each case, the most probable radii of atoms were taken from the reference [67].

## 4. Results and Discussion

Electrophilicity is a conceptual qualitative descriptor useful in the rationale of chemical events.

Since it is a conceptual entity, there is no possibility of its rigorous theoretical derivation. Parr *et al*. [10] suggested an ansatz for evaluating electrophilicity. However, Parr *et al*. [10] seem to have put forward a density functional rationale of their ansatz. Hence *ω* is a density functional descriptor. Thus, so far, the ansatz of Parr *et al*. is the best formula to evaluate *ω* of atoms and molecules. If the *I* and *A* values are reliable, we can set up a reliable bench mark of *ω* values computed through the ansatz of Parr *et al*. [10].

Equation 26 is invoked to evaluate the electrophilicity indices of some selected hetero nuclear di-atomic and polyatomic molecules and the evaluated electrophilicity indices are presented in Tables 1 and 2 respectively.

Since the electrophilicity index has no experimental benchmark, we have made a determined attempt to perform the validity test of our model of electrophilicity equalization in Table 1, where three sets of electrophilicity indices of the di-atomic molecules are presented. We have taken one set of diatomic molecule and another set of tri-atomic molecules and computed their electrophilicity, *ω* in terms of our suggested model and formula. Furthermore, we have invoked the ansatz of Parr *et al*. [10], Equation 2, and computed the electrophilicity indices of the same di-atomic molecules. The required parameters, *I* and *A*, are computed using the *ab initio* quantum chemical method stated above. The electrophilicites published by Chattaraj *et al*. [8] are also presented for sake of comparison.

**Figure 1:**
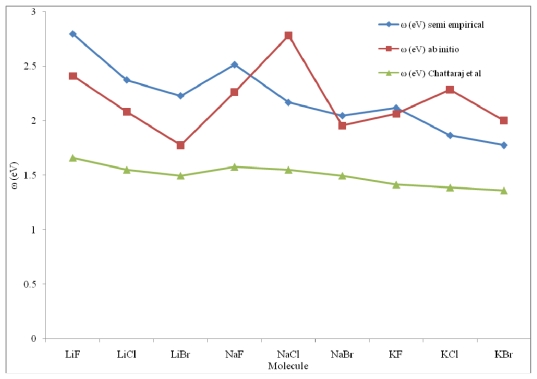
Comparative study of the three sets of electrophilicity indices of some selected hetero nuclear diatomic molecules.

**Figure 2:**
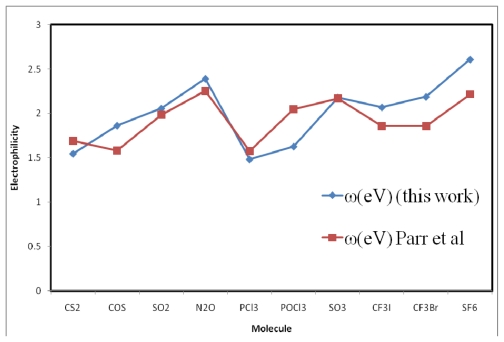
Comparative study of two sets of electrophilicity indices of some selected hetero nuclear poly-atomic molecules.

To perform a comparative study for hetero-nuclear poly-atomic systems, two sets of electrophilicites computed through the ansatz of Parr *et al*. and using experimental *I* and *A*, and those with the formula of the present work for poly-atomic molecules, are presented in Table 2. To have a better view of the comparative study, the results are plotted in Figures 1 and 2.

A close look at Table 1 for di-atomic molecules reveals that the *ω* values computed by the present work are systematically closer to the corresponding values of Parr *et al*. as compared with the corresponding values of Chattaraj *et al*. [8]. Figure 1 reveals that the profiles of electrophilicities of the present work and those of Parr *et al*. [10] are systematically closer and those of Chattaraj *et al*. [8] are far off the profile for the bench mark *ω* values.

Table 2 reveals that there is a strong correlation between the molecular electrophilicity indices of the hetero nuclear polyatomic molecules evaluated through Equation 14, with their corresponding values evaluated using the experimental *I* and *A* through the ansatz of Parr *et al.* [10]. Figure 2 reveals that the profiles of the *ω* values are close and strongly correlated. It is further evident that the *ω* values of as many as four molecular systems are so close that one is almost superimposed upon the other.

## 5. Conclusion

In conclusion we state that we have basically launched a quest as to whether or not there exists a physical process of electrophilicity equalization similar to the phenomena of electronegativity and hardness equalization during the chemical event of molecule formation. The study suggests that the electrophilicity equalization principle is most likely to be a valid theoretical proposition, similar in nature to the electronegativity and hardness equalization principle. We have employed an algorithm invoking the theorem of electrostatics for the computation of the equalized electrophilicity on the event of molecule formation. The results demonstrate that the qualitative view of conceptual chemistry in that there should be a physical process of electrophilicity equalization on the event of molecule formation, is scientifically an acceptable proposition. After a detailed comparative study, it seems that the present model of electrophilicity equalization is an improvement on that of Chattaraj *et al.* [8].

## Figures and Tables

**Table 1:** Computed Electrophilicity index (*ω*) data along with the data of Chattaraj *et al.* [8] of some selected diatomic molecules and the standard deviations of the two sets of data from the values computed using the ansatz of Parr *et al.* [10].

Molecule	*ω* in eV (Present work)	*ω* in eV (Parr *et al.*’s work)	*ω* in eV (Chattaraj *et al.*’s work)	SD in % (Parr *et al.*’s work *vs.* Present work)	SD in % (Parr *et al.*’s work *vs.* Chattaraj *et al.*’s work)
LiF	2.796056041	2.411008	1.66	15.97041739	31.14913
LiCl	2.374237241	2.083325	1.551	13.96384342	25.5517
LiBr	2.230052794	1.776961	1.497	25.49812821	15.75504
NaF	2.515173699	2.263738	1.578	11.10710246	30.29229
NaCl	2.168594646	2.782726	1.551	22.06941517	44.26329
NaBr	2.047669294	1.95519	1.497	4.729938983	23.43455
KF	2.118550154	2.063906	1.415	2.64760866	31.44068
KCl	1.867196746	2.285827	1.388	18.31417049	39.27799
KBr	1.776848516	2.002401	1.361	11.26410165	32.0316

**Table 2:** Computed Electrophilicity index (*ω*) data along with the data computed using the formula of Parr, *et al.* [10] of some selected polyatomic molecules and the standard deviation of the data computed in the present work from the values computed using the ansatz of Parr *et al.* [10].

Molecule	*ω* in eV (Present work)	*ω* in eV (Parr *et al.*’s work)	SD in % (Parr’s work *vs.* Present work)
CS_2_	1.5457	1.69	8.538461538
COS	1.86309	1.58	17.91708861
SO_2_	2.05525	1.985	3.539042821
N_2_O	2.39121	2.257	5.946389012
PCl_3_	1.48275	1.574	5.797331639
POCl_3_	1.62653	2.048	20.57958984
SO_3_	2.17862	2.168	0.489852399
CF_3_I	2.06933	1.857	11.43403339
CF_3_Br	2.18716	1.857	17.77921379
SF_6_	2.60898	2.219	17.57458315
